# Adolescents and young adults with oncohematological disease: use of social networks, impact of SARS-COV-2, and psychosocial well-being

**DOI:** 10.3389/fpsyt.2023.1239131

**Published:** 2023-11-01

**Authors:** Marta Tremolada, Livia Taverna, Francesco Vietina, Roberta Maria Incardona, Marta Pierobon, Sabrina Bonichini, Alessandra Biffi, Gianni Bisogno

**Affiliations:** ^1^Department of Developmental Psychology and Socialization, School of Psychology, University of Padua, Padua, Italy; ^2^Pediatric Hematology, Oncology and Stem Cell Transplant Center, Department of Woman’s and Child’s Health, University of Padua, Padua, Italy; ^3^Faculty of Education, Free University of Bozen-Bolzano, Bolzano, Italy

**Keywords:** cancer, social networking, AYA, communication, reasons for use, social anxiety, COVID19, healthy peers

## Abstract

**Objective:**

Adolescents and young adults (AYA) with oncohematological diseases could have important psychosocial difficulties that could be worsened by the effects of the COVID19 pandemic. At this developmental stage, it is also important to assess the use of social networks (SNs). This study aims to investigate the type of social network use and the consequences of the COVID19 period. These patients are compared with matched healthy peers.

**Methods:**

After the informant consent signature, the adolescents completed a series of self-report questionnaires on the use of SNs, on communication preferences, on social anxiety and on Covid19 impact through the online platform of LimeSurvey. Most of the adolescents belonged to the 18–20 age group (42.5%), were female (62%) and mainly off therapy (72%).

**Results:**

Adolescents spent more than 2 h/day on Instagram and 1 h and half on Whatsapp, while Tik Tok use was on average 1 h/day, especially used by younger patients (*r* = −0.33, *p* = 0.023). Males used Twitch (*t*_45_ = −2.06, *p* = 0.05) and Youtube (*t*_45_ = −2.18, *p* = 0.03) for longer than females. AYA in therapy used more Tik Tok (*U* = 137.50; *p* = 0.03), Ask/Tellonym (*U* = 172.50; *p* = 0.05) and Twitch (*U* = 144; *p* = 0.017) than those off therapy. Healthy AYA showed lower levels of exposure (*Z* = −4.17; *p* = 0.00001) and impact (*Z* = −5.12; *p* = 0.00001) to Covid19, while the level of social anxiety is comparable and is in the normal range in both groups.

**Discussion:**

Some clinical considerations and suggestions could be given based on these empirical results to health professionals in the care of AYA cancer patients.

## 1. Introduction

Adolescence is a complex developmental period in which girls and boys live crucial life moments that host important changes not only from a physical point of view but also from a psychic and social point of view.

The malignant tumor in adolescence is part of a complex phase of life, which hosts various changes not only on a physical level, but also on a cerebral, social, and psychological level. For this reason, adolescents’ patients could show typical pediatric age neoplasms (such as leukemia, brain tumors, sarcomas, or germ cell tumors) and adult-type tumors (such as carcinomas and melanomas) (1).

The Italian Association of Cancer Registries (2) has identified in 2003–2008 years 1,618 new cases of malignancy in adolescents aged 15–19 in Italy and estimates that for the 5-year period 2016–2020 in Italy another 4,000 neoplasms will be diagnosed among adolescents, according to the previous 5-year period. The incidence rate of malignant tumors in adolescents in the period 2003–2008 was 269 new cases per million children per year and is unchanged from the past. Despite the data listed above, the mortality rate is decreasing sharply: in fact, more than 80% of patients with childhood cancer aged 0–19 survive at least five years after diagnosis (3).

Cancer diagnosis in adolescence could impact several aspects: self-esteem (4), identity, autonomy, body image (5), socialization (6), family relationships, psychological health and well-being, also comparing patients with a group of healthy peers (7, 8) or if they have undergone hemotopoietic stem cell transplantation (HSCT) (9).

All the evolutionary changes that adolescents with oncohematological disease have concerning the psychological aspect and the construction of the adolescent’s identity must be inserted within a social context that is constantly evolving and within a civilization that is now immersed in the digital dimension. In recent years, in fact, technological evolution has been sudden and has involved all spheres of adolescents’ life, from the social one, up to the psychological and relational one. Social networks (SNs) become an unavoidable road to take, which has an influence above all on the relational aspect, but also on the social, occupational, and emotional aspects (8).

The motives for the use of SNs in adolescents with cancer are principally: to exchange information on the disease; seek support from people who are experiencing or have experienced the same story; occupy the time; keep in touch with loved ones; share their own personal experience. In fact, the most common research topics concern the causes of the disease and its characteristics, risk factors, symptoms, treatment options, prognosis, and outcome of oncohematologic disease. Furthermore, adolescents may seek information on fertility and sexual health, body image, and exercise physique, nutrition, but also more specific information on support groups online and information on return to school or work (10). AYA with cancer avidly use SNs, noting that they provide a sense of support and community (11).

The literature stressed how it is important to increase efforts on adaptative content to AYA’s cancer in order to help them to know more about their own disease, to increment adherence to treatment and to maintain an important surveillance on post-treatment potential consequences (12). Also AYAs with advanced cancer cited social media and multiplayer online games as a mechanism for maintaining and seeking peer support (13). A number of organizations, many founded by patients and/or their caregivers, have a web and social media presence specifically aimed at AYA patients with cancer.

In addition, physicians and other healthcare professionals are increasingly on social media and interacting with patients and their caregivers (10). When the Covid19 pandemic period began in Italy in February 2020 a new era began, especially for cancer patients and SNs became more frequently used to overcome isolation for the entire adolescent world. Cancer patients who were treated reported greater fear of being infected, greater feeling of personal risk for possible complications, greater feeling of heaviness due to concerns their parents have for their health than their healthy peers (14, 15). In the ordinary adolescent population, it happened: sedentary behavior that increased proportionally to Covid19 screen exposure, decreased physical activity time (16), increased insomnia and sleep-related problems (17) and increased difficulty falling asleep and sudden awakening episodes (18). In addition, from the point of view of psychic and psychological problems, adolescents seem to have been influenced by anxiety symptoms, with some studies showing some influence on suicidal thoughts anxiety levels.

Comparing the ISTAT data for 2019 and 2020, there is an increase percentage of young people aged 15 to 24 who use the Internet (15–17 years 94.7% vs. 95.1%; 18–19 years 93.2% vs. 94.5%; 20–24 years 94.2% vs. 96.2%). There are most report that they use it every day, and even then there is an increase in the percentage of people using the Internet on a daily basis (15–17 years 83.7% vs. 86.8%; 18–19 years 87% vs. 88.2%; 20–24 years 86% vs. 87.2%) (19). The average time spent on social networks is 2 h and 24 min a day. In particular, the data collected in 2020 show how users (13–64 years) spend an average of 23.2 h a month on YouTube, 19.5 h a month on Facebook, 19.4 h a month on Whatsapp, 10.3 h a month on Instagram, 13.3 h a month on TikTok, and 5.1 h a month on Twitch (20). A recent article on Italian adolescents is in line with these parameters (21).

The frequency of use of social network platforms in AYA cancer patients in a recent study (22) identified Facebook (88%), Instagram (62%) and Whatsapp (48%) as the three most popular and used, but they also used other specific online resources to obtain information and support such as national cancer societies, websites, personal blogs and discussion forums.

The three research areas are the following: type of relationship between hospitalized adolescents and the most popular social platforms; the degree of exposure and impact of Covid-19 on AYA cancer patients and the comparisons between these patients and matched healthy peers. See Appendix 1 in Supplementary material for more detailed information.

## 2. Methods

### 2.1. Procedure

The study, therefore, is part of the “stranger teens” project already approved in March 2020 and which sees the involvement of the Pediatric Oncohematology Clinic together with the University of Padua and the non-profit association Team for AYA. The objectives of the aforementioned project concern: the construction of an interdisciplinary care program, designed specifically for adolescents and which can better understand their actual needs; an improvement of the clinical care organization by adapting it to the complexity of the management of these patients, creating a multidisciplinary management, and the evaluation of the actual impact of the project implemented on the quality of life of these patients.

After obtaining the consent of the Psychology Ethics Committee (protocol 4,039, University of Padua), the psychoeducator at the Padua Pediatric Oncohematology Clinic contacted in person AYA in therapy or out of therapy included in the stranger teens program and asked them to participate in the study with prior authorization by filling out the informed consent form (form for adults). For patients under 18 years of age included in the Stranger Teens project, the presentation of the project and the application for membership were made to parents and not directly to AYA.

For the recruitment of participants not included in the “stranger teens” project, recruitment practice was the following: doctors identified patients potentially suitable for participation in the research seeing the databases with their clinic information (i.e., in/off therapy, no presence of significant intellectual disabilities, age range from 12 to 23 years old); they illustrated the objectives of the research, asking for any interest and willingness to participate. If so, they requested authorization to communicate the name / contact to the investigator. The investigator continued with the contact and, subsequently, with the signature of informed consent with handwritten signature and was then scanned and sent to the study director for subsequent administration. Once the signed informed consent has been obtained, the link to the compilation was sent to AYA and parents, which took place online through the LimeSurvey platform. The mean time for filling the questionnaires was about 25 min.

### 2.2. Participants

Table 1 shows the characteristics of the participants. Most adolescents belong to the age group 17–19.

**Table 1:** Sociodemographic characteristics of the participants.

Patients		Controls					
		Frequency	Mean	SD	Frequency	Mean	SD
Gender	Male	18			20		
	Female	29			27		
Age groups	12–14 yrs. old 15–17 yrs. old	9 11	17.53	2.8	10 9	17.5	2.82
	18–20 yrs. old 21–23 yrs. old	20 7			21 7		
Type of job activity	University	12	1.28	0.89	13		
	Job	41			4		
Type of diagnosis	Solid	29					
	Hematological	18					
Status of therapy	In Out	13 34					

The main sociodemographic data collected on the characteristics of the participants were: sex, age range, type of work activity, type of diagnosis, therapy status (see Table 1).

### 2.3. Instruments

#### 2.3.1. SNS time using

To understand how long adolescents thought they would use the social network and the proposed instant messaging system, we were inspired by the work of Pempek & McDaniel (23). We then calculated the frequency of use with a 9-point scale proposed for the estimated frequency of use for Instagram, Facebook, and Whatsapp: i.e., how much time do you spend on Instagram?

1. Never.
2. 10–30 min a day.
3. 31 min – 1 h a day.
4. From 1 h and a half to 2 h a day.
5. From 2 to 3 h a day.
6. From 3 h and a half to 4 h a day.
7. From 4 to 6 h a day.
8. More than 7 h a day.

With this questionnaire, we can have information on the frequency of use of different social networks or instant messaging. These time bands will then also be reduced to average quantities in minutes of use, making it a point variable of an independent type with the other variables of interest in the study.

#### 2.3.2. Motivations for using Facebook

To get an idea of the reasons why teens use Facebook and other social networks, a simplified and adapted version of the Motives to use Facebook (21, 24) was used. The original scale was made up of 20 items, each representing a possible motivation for the use of Facebook, evaluated with a 5-point Likert scale.

In this study, it was decided to adapt the questionnaire to a more generic study context and not just to Facebook and to resize it. The final product was a checklist of 20 items. To not weigh the burden of commitment of adolescents with too many answers, it was decided to set the number of responses for the three main reasons they used social networks, without information on the frequency of choice of the reason for use. However, the teens were left with the freedom to indicate more than three answers, so the selection was anchored in the 3-element checklist and the too selectable answers were limited.

It was also preferred to add another item that allowed the sample to personally write a reason to use social networks in case it was not included in the list provided by the questionnaire.

#### 2.3.3. The social anxiety scale

This scale, obtained from other similar tools, consists of 17 items with a Likert scale 1 (stands for “Never”) – 5 (stands for “Always”) which evaluate three main factors: the fear of a negative evaluation, the social avoidance, distress in the face of the new, social avoidance, and distress more generally. This tool allows you to assess the degree of social anxiety in AYA and then relate it to other aspects such as communication or the use of social media. The minimum score is 20 (very low social anxiety) while the maximum is 68 (very high social anxiety). Cronbach’s alpha calculated on this scale demonstrates good internal consistency of the items (α = = 0.92) (25).

#### 2.3.4. “Tell me how you communicate and I will tell you who you are,” list of communication preferences

This tool has been created *ad hoc* to understand the communicative preferences of AYA in various proposed fields and contexts. The very short questionnaire aims to evaluate the methods preferred by young people to communicate with others on certain topics (21).

Each question (item) corresponded to a sphere of interest (i.e., family, friends, etc.), positive or negative, and a checklist of answers that included 4 different modes of communication and an additional answer in which the participant could write other possibilities.

The items included in each of these selected spheres of interest are the following:

1. Family. 3 items, including 2 negative and one positive.
2. Friends. 2 items, one positive and one negative.
3. School. 2 items, one positive and one negative.
4. Special person. 2 items, one positive and one negative.
5. Other. 3 negative items, one dedicated to teasing, one concerning loneliness, and the last concerning the mood, feeling down.

For each proposed situation, the sample was asked to select which modes of communication they preferred and then which ones they considered most appropriate between:

- Instagram. Representative of the social network category of H.V.S.M. (α = 0.75).
- Facebook. Representative of the classic social network category (α = 0.68).
- Whatsapp. Representative of the verbal instant messaging category (α = 0.87).
- A voice. Generic representative of classical oral speech (α = 0.85).

And more. An option that allowed AYA to write or directly integrate solutions that they preferred, but that were not present in previous answers.

With these questions, we wanted to know between the different areas (1, 2, 3, 4, 5), divided by the type of content (positive or negative), which communication channels were preferred by adolescents (A, B, C, D, E).

#### 2.3.5. COVID-19 exposure and family impact scales – adolescent and young adult version

The COVID-19 Exposure and Family Impact Scales (version for adolescents and young adults) are scales that measure the perceived impact of COVID19 on oneself and their family. They derive from the assumption that the spread of COVID19, which has caused numerous changes in people’s daily lives, was a potentially traumatic experience and caused a worsening of the psychological well-being of the population. Based on this assumption, the greater the influence of COVID19 in the different domains of adolescent and family life, the greater the perceived impact (26).

In the first part, the questionnaire includes 28 items that ask the subject for information about direct exposure to the COVID19 virus. In the second part, there are 14 items that measure the impact of COVID19 on family relationships, on one’s state of mind, and on physical, psychological, and social well-being. The subject can respond by assigning a score ranging from 1 to 4 to each item (1 = Very positive impact, 2 = Very positive impact, 3 = Very negative impact, 4 = Very negative impact). The possibility of adding the item “Not applicable” is also taken into consideration if the pandemic has not caused changes to the domain described in the item. There is then a further question that asks the subject to express from 1 to 10 how much general suffering COVID19 caused (1 = no suffering; 10 = extreme suffering). Finally, there is an open question asking to summarize the positive and negative effects of the COVID19 pandemic experienced by adolescents. The scoring involves calculating the average of the 14 items of the second part to get an idea of the impact caused by the pandemic. Furthermore, it is possible to compare different subjects in the answer given in the third section of the questionnaire. The additional information extrapolated from the last open question can help interpret the subject’s answers. In the present study, only the second and third parts were administered.

### 2.4. Statistical analysis plan

The plan of statistical analyses carried out will now be shown, point by point.

For the first objective, frequencies will be run concerning the daily time spent on social networks – Facebook, Instagram, Whatsapp, Ask/Tellonym, Tik Tok, Youtube, and Twitch – and the reasons of use. To facilitate analysis, it will be decided to transform the measurements of time spent on digital media into a specific variable, transforming each band into its equivalent in minutes.

To respond to the second objective, independent t tests will be shown using the variable of time spent on different digital media as dependent variable, respectively, gender, type of diagnosis group, stage of therapy as independent variables. Pearson or Spearman correlations will be adopted to understand the possible significant association between age and time spent on different digital media.

For the third objective, paired *t*-tests will be performed to understand possible differences between patients and the healthy group.

## 3. Results

The results of research area A followed the hypotheses mentioned in the Appendix 1 in Supplementary material.

### 3.1. Frequency of use of social networks and instant messaging platforms in hospitalized adolescents

The results show that Instagram is the most used platform in terms of daily minutes (101 min), followed by Whatsapp (95 min), Youtube (65 min), Tik Tok (53 min), Twitch (20 min) and finally Facebook and Ask/Tellonym (2.23), which appear to be the social networks least used by adolescents participating in this study (Table 2).

**Table 2:** Frequency of social networking use in hospitalized patients.

	Social network	Mean	SD
Time of use in minutes/day	Whatsapp	95.42	73.18
	Instagram	101.48	74.56
	Facebook	9.25	22.89
	Tik/Tok	52.34	75.19
	Ask/Tellonym	2.23	5.4
	Youtube	65.42	89.5
	Twitch	20.86	53.37

### 3.2. The amount of time spent on social networks by adolescents depends on sex or age

To answer the question about the use of different social networks in relation to the age of patients with AYA, some Pearson’s correlations were carried out showing that as your age increases, the use of Tik Tok gradually decreases (*r* = −0.33; *p* = 0.023), confirming that the main users of this social network are the youngest.

**Figure 1:**
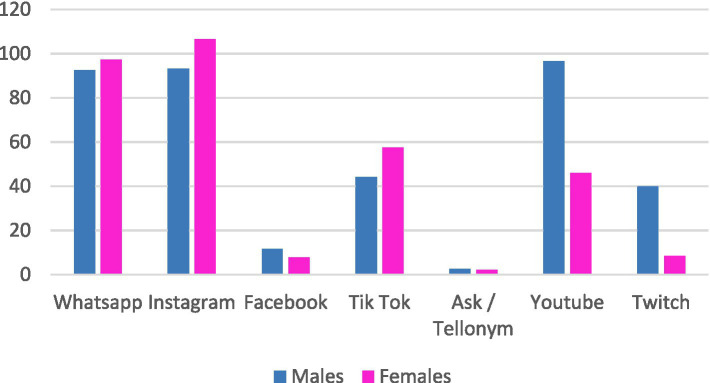
Gender differences in social network use.

We then also wanted to evaluate these frequencies of use in relation to the gender of the participants using the *t*-test for independent samples, to see if males and females make the same use of the platforms or if there may be significant differences. Returning to the reference literature and specifically the work of Riva (27), girls are expected to make more use of Instagram than their male counterparts. To confirm these differences between males and females in terms of frequency of use, two *T*-tests for independent samples were performed alternately inserting time spent on Youtube and Twitch in minutes as the dependent variable and inserting gender as the independent variable. Both Youtube (*t*_45_ = −2.45; *p* = 0.03) and Twitch (*t*_45_ = −2.06; *p* = 0.05) were significantly used differently by sex (Figure 1).

### 3.3. The amount of time spent using social networks depends on the type of diagnosis or the state of therapy

In the same way, the averages related to the daily use of social networks were calculated, dividing them among adolescents diagnosed with solid or liquid cancer. There was no study about this topic, but we decided to explore it to understand better this phenomenon. We expected that patients with longer hospitalizations, treatment cycles and immunodepression status such as those affected by liquid cancers could use less the social media because they could be less motivated to see the positive lives of their friends’ profiles, preferring to stay with the best of them in presence when possible. To study the relevance of the differences between the means, the T-test for independent samples was also carried out in this case, alternately inserting the time spent on social networks in minutes as the dependent variable and inserting the type of diagnosis as the independent variable. Only the difference between the averages of the time of use of Tik Tok between AYA cancer with solid tumor and liquid tumor was found to be significant (*t*_45_ = 2.3; *p* = 0.026). AYA with solid tumors seem to spend more time on Tik Tok and Youtube (respectively 71.38 and 75.52 min per day on average) than those with liquid tumors (21.67 and 49.17 min on average).

Since our participants are representative of the long-term hospital context, the variable linked to the state of therapy was also considered a variable of interest in the study of the averages of the time of use. Proceeding with the Mann–Whitney nonparametric test, the averages relating to the use of Instagram and Whatsapp show no differences to be taken into consideration, in contrast to Tik Tok, Ask /Tellonym, Youtube and Twitch (Table 3). Patients in therapy tend to use both Tik Tok (Mean ranks = 30.42), Ask/Tellonym (Mean ranks = 27.73) and Twitch (Mean ranks = 28.92) more than those out of therapy (respectively Mean ranks: 21.54, 22.57 and 21.36).

**Table 3:** Mann–Whitney non-parametric test between time spent in the different social networks in AYA cancer in or out of therapy.

	Time of use (minutes)						
	Whatsapp	Instagram	Facebook	Tik Tok	Ak/Tellonym	Youtube	Twitch
Mann–Whitney *U*	194	175	179.5	137.5	172.500	152	144
*Z*	−0.67	−1.13	−1.26	−2.1	−1.87	−1.68	−2.38
*p*	0.5	0.26	0.21	0,03	0,06	0.09	0.02

### 3.4. The reasons why cancer patients with AYA use social networks

The best reasons to communicate with AYA patients were boredom (63.8%), communication with friends (51.1%) and staying in touch with them (36.2%), while the less frequent reasons were seeing photos of others (10.6%) or finding information (8.5%).

The three communication preferences most used by AYA in positive or negative situations relating to everyday life or illness (hospital setting) were found to be Instagram, Whatsapp, and the possibility of meeting someone in person to talk. The items were then further divided into two large clusters: the first concerning the positive events and the second concerning the negative ones, so as to be able to have a further key to understanding the communication preferences.

Whatsapp was often used to communicate news such as the good grade received in school (44.7%) or the episode of happiness related to a person you like (46.8%). On the other hand, having fun with friends seems to favor sharing on Instagram (46.8%), while in the medical field, such as sharing the results of post-therapy exams, young people prefer face-to-face dialog (46.8%).

AYA cancer patients seemed to prefer direct confrontation as communication preferences on negative events, i.e., when angry with friends (51.1%), disappointments with friends (51.1%), feeling down (55.3%), communicating about illness (38.3%). For other negative situations related to illness, they reported more adoption of Whatsapp, i.e., boredom in therapy (44.7%) or equal use of Whatsapp and face-to-face, i.e., feeling bad about therapy (36.2%).

### 3.5. Social anxiety levels in patients with patients with AYA cancer and Its significant correlations with variables previously studied

The levels of social anxiety of the participants were calculated using the Social Anxiety Scale for Adolescents (25). As we have seen in the literature (28, 29), it seems that the disease, in hospitalized AYA, can influence anxiety levels. In this study, the average social anxiety was broken down by sex and shows normal values both for females (Mean = 39.34; SD = 13.09; range 20–68) and for men (33.61, SD = 6.87, range 25–51) comparing them with the norms (Females: Mean = 40.46; SD = 12.7; Males; Mean = 37.07; SD = 10.7). Some interesting data were found in relation to social anxiety and the reasons for its use, assumptions that led to the use of the nonparametric Spearman test between these two variables. It was found that higher social anxiety was associated with less use of social networks to send messages to a friend and vice versa (rho = −0.32; *p* = 0.028). However, those with greater social anxiety seem to use social networks above all to kill time and vice versa (rho = 0.3; *p* = 0.042).

The results of research area B followed the hypotheses mentioned above.

### 3.6. Impact of Covid19 on AYA cancer patients

An analysis of the responses of the sample was done in relation to the CEFIS “Impact” scale, which investigated how much and in which areas Covid-19 and the lockdown affected the health and abilities of adolescents. For each item, the scope could be impacted Very Positive (4), Quite Positive (3), Quite Negative (2) or Very Negative (1) and the participant had to report it. Looking at the results, Covid-19 seemed to have a very or quite negative impact on the family climate (68.10%), on the ability to take care of one’s health (76.60%), on independence skills (61.70%) and on that of taking care of others (68.10%). Furthermore, looking at the items related to physical well-being, we can see that a good percentage of the participants in the clinical group negatively rated the impact of the pandemic on the diet (42.60%) and the ability to sleep well (68.10%). These data confirm the results of other studies (17, 18) on worsening sleep quality during the pandemic period. As far as emotional well-being is concerned, two interesting data are those related to anxiety/fear and loneliness. In both items, there are significant percentages of impact of Covid19 on these issues: 46.8% for anxiety/fears and 51.10% for loneliness.

### 3.7. Relationship between the impact or exposure to Covid-19 and social anxiety, communication preferences, or reasons for using social networks

Various exploratory correlations were made considering mean social anxiety and other variables related to the CEFIS scale (particularly exposure and impact). We run Spearman’s correlations and found that as social anxiety increased, Covid19 exposure (rho = 0.36; *p* = 0.014) and impact (rho = 0.44; *p* = 0.003) values were also higher and vice versa. Therefore, those who were more exposed to Covid-19 and reported a greater impact also appeared to have higher levels of social anxiety.

Other interesting data was obtained between impact and communication preferences. In fact, there were several significant correlations: the higher the perceived impact of Covid19 impact from adolescents, the greater their propensity to share *via* Instagram the last day of therapy (rho = 0.31; *p* = 0.039), to use this social network when bored during therapy (rho = 0.45; *p* = 0.002) and to share aspects related to their disease (rho =0.33; p = 0.03). At the same time, since the Covid19 perceived impact of Covid19 was higher reported, the need to meet in person with someone to share the results of the exams also significantly increased therapy (rho = 0.32; *p* = 0.035) and talk about the disease itself (rho = 0.42; *p* = 0.005). Taking into account the reasons for use, the patients who were the most exposed and most affected by Covid had a low propensity to use social networks to send messages to a friend (rho = −0.50; *p* = 0.00001).

The results of the research area C followed the above-mentioned hypotheses.

### 3.8. differences in the timing of use of social networks in AYA between the clinical and control groups

It was decided to compare the collected data relating to the hospitalized child group with those of the control group, with respect to the use of social networks, social anxiety, and exposure/impact of Covid-19. The 47 healthy peers were selected and matched with those in the clinical group based on similar sociodemographic characteristics (age, siblings, school).

### 3.9. Difference in communication preferences between the clinical and control groups

To investigate any significant differences between these two groups, it was decided to use the nonparametric Wilcoxon test for paired samples. Hospitalized adolescents used Ask/Tellonym more frequently (Z = −2.121; *p* = 0.034; Mean = 2.23; SD = 5.39) and Facebook (Z = −1.882; *p* = 0.06; Mean = 9.25; SD = 22.9) than healthy peers (specifically for Ask/Tellonym: Mean = 0.32; SD = 2.19; for Facebook: Mean = 2.55; SD = 7.86).

### 3.10. Difference in the levels of social anxiety, exposure, and impact of Covid-19 between the clinical and control group

**Figure 2:**
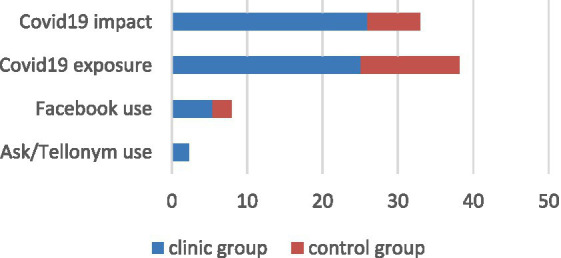
Significative differences between the clinic and control groups.

The two groups were also compared social anxiety and the results of the CEFIS scales, both exposure and impact. Wilcoxon tests for paired samples were performed. Of the two groups, the control group reported lower levels of exposure (*Z* = −4.169; *p* = 0.00001; Mean ranks = 13.8) and impact of covid (*Z* = −5.123; *p* = 0.00001; Mean ranks = 7) compared to the group of AYA with an oncology disease (respectively Mean ranks for exposure = 25.06; Mean ranks for impact = 25.94). These data seem to confirm the results of the study by Casanova et al. (14), in which adolescents in therapy showed greater fear of being infected and greater sense of personal risk of possible complications. However, in terms of social anxiety, no significant differences were found between the two groups (Figure 2).

## 4. Discussion

Following the first research area, we decided to outline the times of use, the communication preferences, and the reasons for using these social platforms. The data presented in the literature suggested a continuous growth in the use of social networks and digital media in the lives of not only adolescents; the precedent studies offer a ranking that sees the instant messaging application as the first placed (Whatsapp, 90.2%), followed by Facebook (88.3%) and Instagram (54.5%) (19). More recent research confirms this classification by updating the Italian usage percentages (Whatsapp 84%; Facebook 81%; Instagram 55%) and specifying the average daily use of social networks as a whole, 1 h and 51 min per day (20). These data were only partially confirmed by the results of our study: In fact, although Whatsapp was used on average 95 min a day, Facebook use seemed to be declining among the new generations of young people (only 9 min per day). The social network used most was Instagram (101 min a day), in disagreement with the literature, as well as the rise of Tik Tok, which in the last 2–3 years has seen an exponential increase not only in members, but also the use (52 min a day). The results of our study confirmed a significant association between age and the use of the latter platform: the older the age of the participants, the less Tik Tok use. Youtube also seems to be used extensively by teenage boys (65 min a day). However, we have to consider that Global Digital (20) studies analyzed estimates of the entire Italian population without specifying the percentages of use based on age group. This could be a first cause behind the differences with the results of our study. In general, there seemed to be an increase in the use of “fast” platforms, such as instant messaging social networks, and which have images/videos, such as Instagram, Tik Tok, and Youtube with an average of almost an hour of use per day. These data outline one of the distinctive traits of today’s adolescents that of “communication speed,” the need to communicate, see, learn and share through images and videos. They are interested more in what is visually impactful, rather than something written. Social media provides a promising platform for reaching and engaging both other AYAs and their precedent social network. For this reason, they have a very high rate of usage, with the majority of individuals aged 12–25 using them. A variety of social media platforms can provide networking and support opportunities to patients, allowing them to dampen their difficulty fitting in with friends and experience loneliness. However, these online activities should be monitored by health professionals both for scientific validity of their medical contents and also to avoid possible bullying victimization or anxiety symptoms.

In addition to age, gender also appears to be a particularly influential variable. Returning to the literature, and specifically the work of Riva (27), women were expected to make more use of Instagram than men. This trend was confirmed by the results of our research (with 106 min per day for women versus 93 min for men), even if the difference was not particularly significant. On the contrary, a substantial difference in along gender was discovered regarding the Youtube platforms (96 min a day for men and 46 for woman) and Twitch (40 min a day against 8 min). These results can be contextualized by thinking about the greater use men make of video games compared to women. In fact, on both Youtube and Twitch, it is possible to watch videos streamed by other users while they play their video games. These data accompany the advent into the digital world of youtubers and gamers, guys who manage channels with thousands of subscribers by offering live broadcasts and the videos they record while playing.

Since the participants in this study are representative of hospitalization in a long-term setting, we were also interested in studying the times of use in relation to the state of therapy and the diagnosis of the tumor. In this case, we do not have exhaustive studies that have investigated the relationship between these variables. However, an interesting fact that emerged from our study is that the adolescents in therapy used more Tik Tok, Ask/Tellonym and Twitch compared to their peers out of therapy. This difference may be due to boredom and the longer waiting times and “empty moments” experienced by young people undergoing therapy. At the same time, the data regarding the use of the Ask/Tellonym platform is interesting. This social network is known precisely for its characteristic that differentiates it from all the others: the possibility of asking questions and answering them in total anonymity. The fact that teenagers in therapy use it significantly more makes us reflect on how the possibility of anonymity can be a useful tool for them, a sort of refuge, an opportunity to chat and share thoughts without having to reveal one’s name or image. As already mentioned in the first chapter, the physical appearance and identity of these boys are challenged not only by the typical changes of their transitional age but also by the trauma of the disease and the side effects of the therapies.

Regarding the reasons for use, the main motivation that emerges from the study, that of boredom, is particularly interesting. This fact can open some avenues for reflection regarding our young people today, who seem increasingly dependent on time, constantly looking for something that can fill their hours, even passively. However, let us remember that our group of participants represents a hospital reality that is characterized by visits, dead times between one exam and another, and, especially during the Covid-19 period, a lot of solitude. In this framework, the use of social networks as “fillers” or usable as a weapon to combat boredom acquires a different connotation. This perspective that sees social networks as a sort of “escape” from boredom is further confirmed by the results of studies on social anxiety. In fact, adolescents who show high social anxiety appear to use social networks especially to save time.

Some studies have highlighted the negative impact of the pandemic and the closure on the physical well-being of AYA. In particular, previous studies (17, 18) have highlighted how the pandemic has had a negative impact on sleep quality, while others (16, 30, 31) focused on the increase in sedentary behaviors. The results of our studies confirm these hypotheses: in fact, the boys reported a negative influence not only on these two aspects (sleep 68.10%, sedentary behavior 51.10%), but also on the family atmosphere (68%), on the ability to take care of one’s own health (76.6%), and that of others (68.10%).

From the results, it can be seen that the boys who show high levels of social anxiety are those who at the same time report a high exposure to Covid-19 and that they feel that this has had a major impact on their lives. We can interpret this result to mean that adolescents with high social anxiety are probably more fragile and consequently more susceptible to the negative effects of the pandemic or to how this isolation condition in which they have been forced to live has also increased their social anxiety.

Regarding the last research area on possible differences between patients and healthy peers, a first difference was found in the extent to which it concerns the times of use; in fact, hospitalized AYA seem to use both Facebook and Ask/Telonym more. The use of this anonymous platform, which is increasingly used by hospitalized AYA, reconfirms the hypothesis that anonymity could be a useful communication dynamic for them. The other interesting data point that prompts us to reflect is that concerning communication preferences: Healthy peers, in fact, use significantly more Instagram and Whatsapp to share both positive and negative events with respect to patients. As we have seen, these two social networks are by far the most used, but these results lead us to have to differentiate the type of use between active and passive. Returning to the results on the reasons for use, very often social networks play a “filler” role, a gaze passive, without necessarily having to share or interact. This type of use is a passive use, and we have seen above how it characterizes the especially the days of hospitalized adolescents, who have to cope with numerous moments of waiting and boredom. The latter result presented instead underlines how adolescents in the control group, compared to hospitalized ones, use social networks in an active way, which leads them to share some aspects of their lives more on digital platforms.

A final interesting comparison data is the one concerning the exposure and the impact of Covid-19 between the two groups. In fact, adolescents in the control group reported significantly lower levels of exposure and impact compared to those of the clinical group. Also, in this case, the disease seems to lead to a sort of “fragility.”

The present study addressed very recent issues; for this reason it was not able to enjoy an exhaustive literature that would have allowed for some interesting large-scale comparisons. The topics of interest provide for a constant updating of the results due to the frequent and sudden sociotechnological changes characteristic of the current period.

In addition to the limited number of participants in our clinical group, even its composition appears to be unbalanced, both from the point of view of age, which sees a greater number of subjects belonging to the 18–23 age group, but also from the point of view of gender, females are more numerous than males (29 against 18). A further limitation objective of this work is represented by the levels of social desirability: Being all the proposed self-report questionnaires it is easy to run into this type of difficulty, thus influencing the results.

As far as future prospects are concerned, however, at an application level, one could think of offering hospitalized AYA some meetings where they could be protagonists in the use of social networks in an educational and pro-active way. Some sharing meetings could also be organized to address, in addition to the issue relating to social networks and their reasons for using them, also some issues relating to the pandemic and the “return to normality,” perhaps by bringing them together with some experts of the sector.

## 5. Conclusion

This study allows us to get closer to the current reality in which today’s young people are immersed and to know their habits, preferences, and consequences experienced by hospitalized adolescents in response to exposure and the frequent interaction with the social world. The results obtained suggest to us how much this new reality is an integral part of everyday life and allow us to understand its influence on their daily lives. In addition to this, it was possible to investigate their personal experiences in this pandemic reality, trying to highlight the areas of life and development that have been more or less affected.

Their physical, emotional, and psychological well-being remains a complex reality, full of enthusiasm, hard work, and research, within which the pandemic has broken out, leading the adolescents to face a further challenge, dosing their energies, depleting them in.

What is certain is that the consequences and repercussions will be seen in the years to come. For this, it will be appropriate to consider critical issues in a comprehensive manner together with the possibilities and prospects, to be able to keep pace with the continuous revolutions and to educational adapt both the hospital and the school worlds to the latter.

The goal is, therefore, to encourage and enhance the continuous updating of the specific approaches of the area considered up to now, without, however, forgetting the past achievements and perspectives, to be able to “keep up” with the dynamics of change rapid and constant and the positive and negative effects that these generate in development psychological, social, and emotional of adolescents, even in hospital care.

The clinicians should be trained to become more confident with this new social media phenomenon, using and adapting it for their caring objectives. Health professionals should consider social media as another new way of communication with and between patients, closer monitoring their possible feelings, doubts, and questions. This way of communication should not substitute the classical one in presence, but both two could be used and possible support intervention program could be set up using the social media as a resource not a phenomenon to be condemned. Social media can play an important role in facilitating social support for AYAs and enabling AYAs to overcome barriers associated with traditional, in-person support groups (e.g., transportation, time, energy). Social media can also connect patients with similar diagnoses or help maintain friendships formed during treatment (32). However, some patients could report cancer-related cyberbullying (11) and connecting with peers through social media may also amplify feelings of frustration and anxiety (33). Health clinicians must enhance positive and blunt negative Social Media interactions by addressing and guiding their use in AYA patients.

## Data availability statement

The original contributions presented in the study are included in the article/ Supplementary material, further inquiries can be directed to the corresponding author.

## Ethics statement

The protocol was approved by the Psychology Ethics Committee, University of Padua (protocol 4039). All subjects gave written informed consent for participation in accordance with the Declaration of Helsinki.

## Author contributions

MT and SB: conceptualization and methodology. MT and FV: formal analysis. FV: investigation. MT: data curation, writing—original draft preparation, supervision, and project administration. AB and GB: resources. MT and LT: writing—review and editing. MP and GB: visualization. All authors have read and agreed to the published version of the manuscript.
