# Supplementary material for: Adolescents and young adults with oncohematological disease: use of social networks, impact of SARS-COV-2, and psychosocial well-being
Source: Front Psychiatry. 2023 Nov 1;14:1239131. doi: 10.3389/fpsyt.2023.1239131 (PMC10646548; doi:10.3389/fpsyt.2023.1239131)
Supplement: Supplementary file 1 [file Data_Sheet_1.docx]

Supplementary Material

Adolescents and young adults with oncohematological disease: Use of social networks, impact of SARS-COV-2 and psychosocial well-being

Marta Tremolada^*^, Livia Taverna, Francesco Vietina , Roberta Maria Incardona, Marta Pierobon, Sabrina Bonichini, Alessandra Biffi, Gianni Bisogno

*** Correspondence:** Corresponding Author: marta.tremolada@unipd.it

# Appendix 1: research areas and specific hypothesis

# The first research area (A) investigates the type of relationship between hospitalised adolescents and the most popular social platforms.

# H1: How many hours do hospitalised adolescents spend a day on social networks and instant messaging platforms?

# H2: Does the amount of time and the mode spent using social networks change depending on the gender or the age?

# H3: Does the amount of time spent using social networks change depending on the type of diagnosis or the state of therapy?

# H4: What are the reasons for using social networks in AYA cancer?

# H5 What are the levels of social anxiety in patients with AYA cancer? Are there significant correlations between this parameter and the variables studied variables?

# The second research area (B) focuses on the degree of exposure and impact of Covid-19 on AYA cancer patients.

# H1: What kind of impact has Covid-19 had on the lives of patients with AYA cancer?

# H2: Is there a significant relationship between the impact or exposure to Covid-19 and social anxiety, communication preferences, or the reasons for using social networks?

# The third area of investigation (C) deals with the comparison between patients with AYA cancer and matched healthy peers.

# H1: Is there a significant difference in the timing of use of social networks in AYA between the clinical and control groups?

# H2: Is there a significant difference in communication preferences between the clinical and control groups?

# H3: Is there a significant difference in the levels of social anxiety, exposure, and impact of Covid-19 between the clinical and control groups?
